# Shoulder Instability in the U.S. Military: A Systematic Review of Epidemiology, Operative Management, and Outcomes

**DOI:** 10.3390/jcm15010110

**Published:** 2025-12-23

**Authors:** John R. Tyler, Hunter Czajkowski, Alexis B. Sandler, Nicholas M. Brown, Dane Salazar, John P. Scanaliato, Jonna Peterson, Nata Parnes

**Affiliations:** 1 Department of Orthopaedic Surgery & Rehabilitation, Loyola University Health System, Maywood, IL 60153, USA; johntyler861@gmail.com (J.R.T.);; 2 Department of Orthopedic Surgery, Carthage Area Hospital, Carthage, NY 13619, USA; h6czajkowski@gmail.com; 3 Department of Orthopaedic Surgery and Rehabilitation, William Beaumont Army Medical Center, Texas Tech University Health Sciences Center El Paso, El Paso, TX 79905, USA; 4 Health Sciences Library, Loyola University of Chicago, Maywood, IL 60153, USA

**Keywords:** shoulder dislocation, glenohumeral instability, military personnel, posterior instability, glenoid bone loss, incidence

## Abstract

**Background:** Shoulder instability imposes a substantial burden in U.S. military populations, yet epidemiology and outcomes reporting is heterogeneous. This study aims to quantify the epidemiology of shoulder instability among U.S. active-duty servicemembers and to report operative management patterns and outcomes. **Methods:** A systematic review was performed by searching MEDLINE, EMBASE, Scopus, Cochrane, and SPORTDiscus through 1 August 2025. Eligible studies enrolled U.S. active-duty servicemembers with clinical and/or radiographic evidence of instability. After a single comprehensive search with uniform inclusion criteria, studies were assigned to two prespecified cohorts: (1) epidemiology (incidence, directionality, risk factors) and (2) operative management/outcomes (procedure distribution, failure, complications, return to duty [RTD] and return to sport [RTS]). Incidence was pooled as a person-years–weighted fixed-effect estimate; directionality proportions were meta-analyzed with random-effects (logit-transformed) models among patient-level, unidirectional cases. **Results:** Forty-nine studies were included (epidemiology, *n* = 8; outcomes, *n* = 41). Three epidemiologic datasets (42,310 events; 20,472,363 person-years) yielded a pooled military incidence of 2.07 per 1000 person-years (95% CI, 2.05–2.09). Among unidirectional cases (*n* = 916 shoulders), anterior instability comprised 83.9% (95% CI, 70.5–91.9) and posterior the remaining 16.1% (95% CI, 8.1–29.5). Outcome series most commonly reported arthroscopic Bankart repair (*n* = 933 shoulders), bony augmentation (e.g., Latarjet/Bristow; *n* = 700), posterior labral repair (*n* = 649), combined repairs (*n* = 511), and open Bankart (*n* = 442). Weighted mean failure ranged 4.7–23.6%; complications 5.2–10.9%; and reoperations 5.3–17.7%. RTD ranged 50.0–84.7% and RTS 4.8–75.0%. **Conclusions:** Shoulder instability in U.S. servicemembers occurs at rates exceeding population-based civilian estimates, with a relatively greater share of posterior and combined patterns. Operative outcomes vary substantially across procedures.

## 1. Introduction

Shoulder instability is a major source of morbidity in young, physically active populations and is particularly burdensome in the U.S. military, where regular high-volume training and physical fitness testing, as well as occupational contact activities and overhead tasks, create sustained exposure to traumatic and repetitive stress as a result of high occupational shoulder demand [1,2,3,4]. Large military surveillance studies consistently show higher instability risk in servicemembers as compared to civilians, with demographic and service-related factors repeatedly associated with injury [5,6,7,8].

Patterns of pathology in military cohorts also diverge from civilian series. Posterior and combined-type (anterior–posterior) instability are relatively more frequent, and symptomatic subluxation accounts for a substantial share of clinically meaningful instability, findings replicated across service academies and major military branches [1,9,10].

Surgical indications for stabilization in this population arise from the interplay between clinical presentation (frequency and severity of instability events, subjective apprehension, pain, and occupation- or sport-specific demands) and the underlying structural pathology, including instability direction, glenoid and humeral bone loss, capsulolabral injury, and long-head-of-biceps involvement. These patient- and lesion-level factors not only guide selection among available procedures but also strongly influence postoperative failure and revision risk [11,12].

Operative management options span from soft-tissue stabilization (arthroscopic/open Bankart repair, with or without remplissage; posterior or combined labral repair; capsular plication) to bony augmentation (e.g., Latarjet/bone-block) [13,14,15,16,17]. Reported failure, complication, and reoperation rates vary widely by procedure, and functional recovery is inconsistently captured. Return to duty (RTD) and return to sport (RTS) rates are variably defined and timed, and recurrence is reported with nonuniform follow-up horizons and thresholds, complicating between-study comparisons and evidence-based counseling.

Despite a growing body of military-specific research, the literature remains fragmented across epidemiology, directionality, and operative outcomes, and reporting often lacks standardized denominators and uniform outcome definitions. A focused synthesis dedicated to active-duty populations is needed to contextualize systemic burden, clarify patterns of pathology and treatment, and identify gaps that should guide prospective study design and clinical decision-making.

To address this need, we conducted a systematic review of shoulder instability in U.S. servicemembers with the goal of integrating evidence across two prespecified outcome domains: (1) epidemiology (incidence, directionality, risk factors) and (2) operative outcomes (procedure distribution, failure/recurrence, complications, RTD, RTS). We hypothesized that the incidence of shoulder instability in military cohorts would exceed published civilian benchmarks, that posterior instability would represent a greater share than typically reported in civilians, and that postoperative outcomes would vary according to the type of procedure performed.

## 2. Methods

This systematic review was conducted according to the PRISMA (Preferred Reporting Items for Systematic Reviews and Meta-Analyses) guidelines with checklist compliance provided in the Supplementary Materials and prospective registration (PROSPERO CRD420251120478) [18].

### 2.1. Search Strategy

Two independent reviewers (J.R.T., H.C.) searched MEDLINE (PubMed), EMBASE (Elsevier), Scopus (Elsevier), Cochrane Library, and SPORTDiscus (EBSCO) from database inception through 1 August 2025. Scopus and Embase were included to enhance coverage of new and emerging literature. Search strategies combined keywords and controlled vocabulary for shoulder instability and joint instability (anatomic qualifier: shoulder) and included terms specific to military personnel. The complete database-specific strings and coverage are provided in Supplementary Materials.

### 2.2. Study Selection

Studies were eligible for inclusion if they examined U.S. active-duty servicemembers with clinically and/or imaging-confirmed shoulder instability. Eligible designs included retrospective or prospective cohorts, case-control studies, and case series with extractable group-level data. Exclusion criteria were: studies of civilians without a military subgroup; non–U.S. military populations; populations exclusively <18 years; case reports, narrative reviews, editorials, conference abstracts without full data; and studies lacking extractable group-level data. All studies were classified as Level 4 evidence or higher.

**Figure 1:**
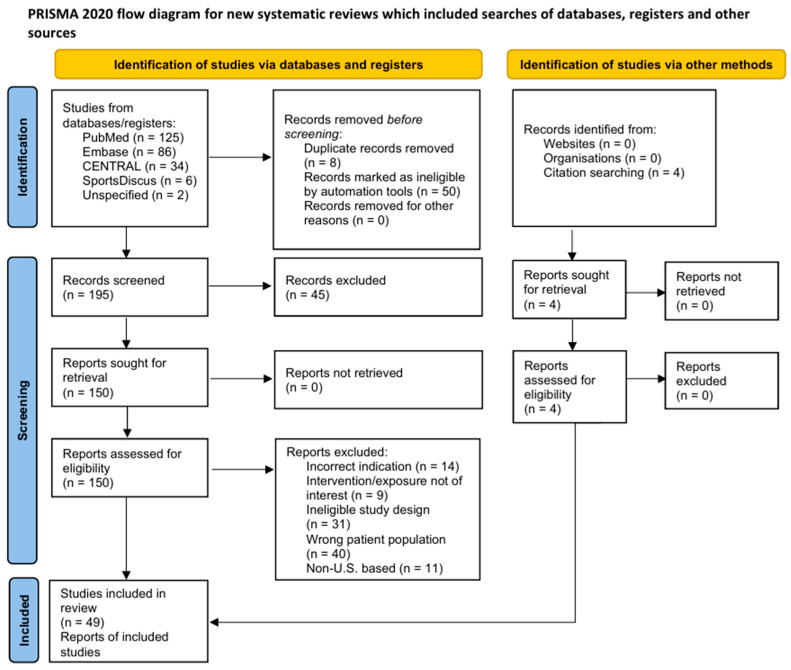
PRISMA (Preferred Reporting Items for Systematic Reviews and Meta-analyses) flow diagram.

Titles and abstracts were screened independently by both reviewers, followed by independent full-text review of potentially eligible records. Discrepancies were resolved by discussion and, when necessary, consultation with a third reviewer (N.P.). Reasons for full-text exclusion were recorded and are summarized in the PRISMA flow diagram (Figure 1). Following inclusion, each study was assigned to one of two mutually exclusive prespecified analytical cohorts: (1) epidemiology or (2) operative management/outcomes.

### 2.3. Data Extraction and Operational Definitions

For epidemiologic studies, extracted variables included incidence numerators and denominators as events and person-years, demographics (e.g., age, sex), instability characteristics (e.g., mechanism and directionality), and reported risk factors. Risk factors were considered “significant” when the source study reported *p* < 0.05 or a 95% confidence interval (CI) excluding 1.0; due to heterogeneous covariate definitions and models, these were summarized descriptively rather than pooled. Civilian reference values were abstracted for context but were not included in pooled estimates.

For postoperative outcomes studies, extracted values included procedure type(s), approach (arthroscopic vs open where applicable), the cumulative number of shoulders per technique, and outcomes data. RTD was operationally defined as return to unrestricted full duty at the same or higher level of responsibility, and RTS as return to the preinjury (or higher) sport level. Failure was defined as recurrent instability (dislocation/subluxation) or reoperation for instability.

Procedures were classified a priori as primary stabilization when performed with the intent to restore glenohumeral stability, including Bankart repair (arthroscopic and open), isolated posterior labral repair, capsular shift/plication, and bony augmentation procedures (Latarjet, Bristow, or Eden–Hybinette). Concomitant procedures were defined as additional interventions performed during the same operative episode that were not intended to directly correct instability, including SLAP repair (types II–IV or combined lesions), rotator-cuff procedures (repair or debridement), and acromioclavicular joint procedures (distal clavicle excision, resection arthroplasty, or reconstruction). In this taxonomy, remplissage was analyzed as a concomitant augmentation rather than a primary stabilization procedure. Studies in which stabilization or labral work was incidental to another primary indication (e.g., rotator-cuff repair) were excluded from primary-stabilization counts.

### 2.4. Methodological Quality Assessment

The methodological quality of included nonrandomized studies was assessed using the Methodological Index for Non-Randomized Studies (MINORS) criteria [19]. This validated instrument evaluates 8 items for noncomparative studies and 12 items for comparative studies, with each item scored as 0 (not reported), 1 (reported but inadequate), or 2 (reported and adequate), yielding a maximum score of 16 for noncomparative studies and 24 for comparative studies.

Each included study was independently reviewed and scored according to the MINORS criteria based on study design, reporting quality, and methodological rigor. Comparative studies were evaluated using the additional four comparative-specific domains. Discrepancies in scoring were resolved by consensus discussion among reviewers. The results of the methodological quality assessment are summarized in Table 1, which presents item-level scoring and total MINORS scores for all included studies.

### 2.5. Data Synthesis

For the epidemiology cohort, two prespecified quantitative analyses were performed. First, shoulder instability incidence rates in the military population reported per 1000 person-years were pooled using a person-years–weighted fixed-effect average; study-level 95% CIs were computed with exact Poisson methods. Given that only three surveillance studies were eligible and case definitions differed, between-study heterogeneity and prediction intervals were not estimated. Second, directionality was synthesized only among patient-level, unidirectional cases. Cohorts were eligible if they reported the proportion of anterior versus posterior instability within the same denominator of unidirectional cases. Event-level reports, one-sided reports, and combined or multidirectional patterns that could not be disaggregated were excluded. For each eligible study, within-study proportions were recalculated so that anterior + posterior = 100% of unidirectional cases. Pooled proportions were estimated with a random-effects model (logit-transformed proportions; inverse-variance weighting), with study-level CIs via exact binomial methods. A prespecified sensitivity analysis repeated the directionality meta-analysis including the single operative-only military cohort to assess case-mix effects. Leave-one-out influence analysis was also conducted.

Studies reporting only posterior cases or event-level (rather than patient-level) data were excluded from pooling and summarized descriptively. Combined (anterior–posterior) and multidirectional instability were variably defined and reported, and therefore synthesized qualitatively.

Outcomes studies were synthesized qualitatively and, when eligible, contributed to procedure-specific pooled outcomes. Procedure-specific outcomes were pooled as proportions only when studies reported both the numerator and denominator at the procedure level (e.g., failures per shoulders treated). Mixed-procedure cohorts that did not segregate failure counts by procedure were excluded from procedure-level pooling and retained only in qualitative summaries, and four studies were excluded from procedure-level pooling for this reason.

### 2.6. Statistical Analysis

Incidence analyses reported study-level rates with exact Poisson 95% CIs and a person-years–weighted pooled incidence (fixed-effect; heterogeneity metrics not calculated). Directional distribution analyses reported pooled proportions with exact binomial 95% CIs using a random-effects model. I^2^ and τ^2^ were reported for these random-effects analyses and interpreted cautiously given small k. For other outcomes, including recurrence, RTD, and procedure-specific functional measures, heterogeneity in study design, outcome definitions, and follow-up precluded formal comparative meta-analysis, so descriptive summaries are presented. Continuous variables are presented as means with standard deviations and ranges, and when per-study sample sizes were available, weighted means were calculated. All statistical analyses were performed using R version 4.4.1 (R Foundation for Statistical Computing, Vienna, Austria).

## 3. Results

The search identified 257 records (databases/registers, *n* = 253; other sources, *n* = 4). After removing duplicates (*n* = 58), 199 titles/abstracts were screened, 154 full texts were reviewed, and 49 studies included (epidemiology, *n* = 8; operative management/outcomes, *n* = 41). No records were unretrieved (Figure 1).

### 3.1. Epidemiology Cohort

#### 3.1.1. Incidence of Shoulder Instability

**Figure 2:**
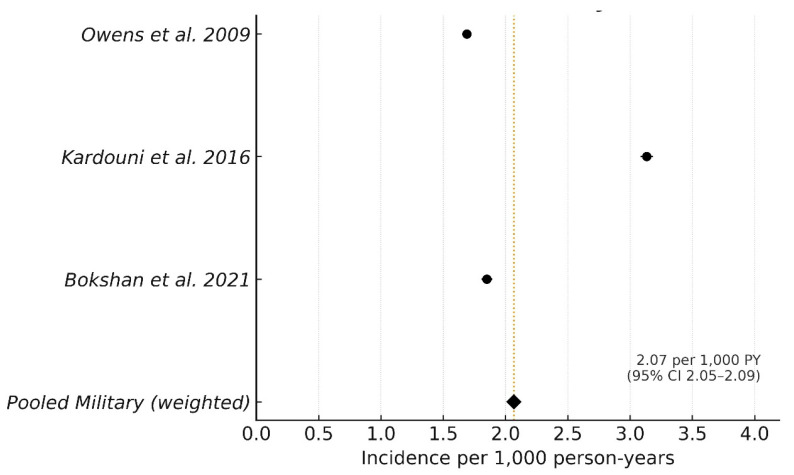
Forest plot of the incidence of shoulder instability in active-duty U.S. service members. Individual study rates are shown with 95% confidence intervals; marker sizes are proportional to person-years. The pooled military incidence (diamond) is a person-years–weighted fixed-effect estimate of 2.07 per 1000 person-years (95% CI, 2.05–2.09). A vertical dotted line indicates the pooled rate. CI = Confidence Interval [2,21,38].

Three epidemiologic studies were eligible for quantitative pooling [6,21,38]. Baseline characteristics of these epidemiologic cohorts are summarized in Table 2. Across these studies, 42,310 instability events occurred over 20,472,363 person-years of active-duty service. Individual study incidences ranged from 1.69 to 3.13 per 1000 person-years (Table 3). The pooled weighted incidence of shoulder instability in the U.S. military was 2.07 per 1000 person-years (95% CI, 2.05–2.09; Figure 2). This rate was higher than population-based civilian estimates (0.24 per 1000 person-years) and collegiate-athlete estimates (0.12 per 1000 athlete exposures), though denominators and case definitions differ and were not pooled with military data [5,8].

#### 3.1.2. Demographics and Risk Factors

Five studies reported patient demographics and risk factors (Table 4) [6,7,21,22,38]. Mean age ranged from 18.8 ± 1.0 to 22 ± 4 years and males comprised 86–96% (median, 88%). Across studies, male sex, younger age, and White race were consistently associated with higher incidence or recurrence. Lower rank emerged as an independent risk factor in multiple cohorts, and prior instability and concomitant axillary nerve injury predicted recurrence when reported [22,38].

#### 3.1.3. Directional Distribution

**Figure 3:**
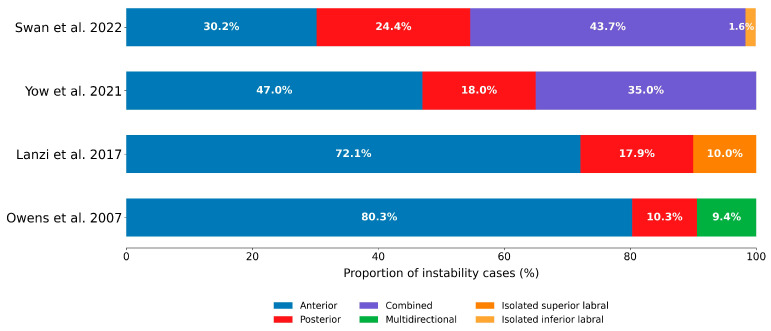
Distribution of instability direction categories by cohort in U.S. military populations. Bars show the proportion of cases classified as anterior, posterior, combined (anterior–posterior), or multidirectional; where reported, additional categories (e.g., isolated superior labral) are displayed [1,7,10,39].

Combined (anterior–posterior) instability was reported in two cohorts, representing 34.5–43.7% of cases within those studies, and multidirectional instability was reported in only one cohort (9.4%). The full distribution of instability direction categories by cohort is presented in Table 5 and Figure 3.

Among epidemiology cohorts reporting both directions (k = 3; *n* = 916 unidirectional cases), anterior instability comprised 83.9% (95% CI, 70.5–91.9) and posterior 16.1% (95% CI, 8.1–29.5); heterogeneity was substantial (I^2^ = 93.9%) and should be interpreted cautiously given small k. In a prespecified sensitivity analysis that included the outcomes-only cohort, pooled anterior instability accounted for 78.4% (95% CI, 60.8–89.5), an absolute decrease of 5.5 percentage points relative to the epidemiology-only analysis, with I^2^ = 96.5%. Leave-one-out analysis identified the U.S. Naval Academy cohort as the principal source of heterogeneity; excluding Yow et al. (2021) yielded anterior 88.0% (95% CI, 85.2–90.3) with I^2^ = 0% [7]. Distributions of anterior versus posterior instability among unidirectional cases across cohorts are summarized in Table 6.

#### 3.1.4. Recurrence

Two studies reported recurrence characteristics (Table 7). Owens et al. (2007) conducted cross-sectional surveillance over a single academic year and found that recurrent events constituted 54.5% of subluxations and 33.3% of dislocations at presentation [1]. Kardouni et al. (2016) reported a 2-year recurrence rate of 28.7%, defining recurrence as a repeat dislocation or instability episode occurring ≥3 months and ≤2 years after the index event [38]. Of the epidemiological studies, none reported recurrence characteristics at over two-year follow-up timepoints.

### 3.2. Operative Management/Outcomes Cohort

#### 3.2.1. Procedure Distribution

**Figure 4:**
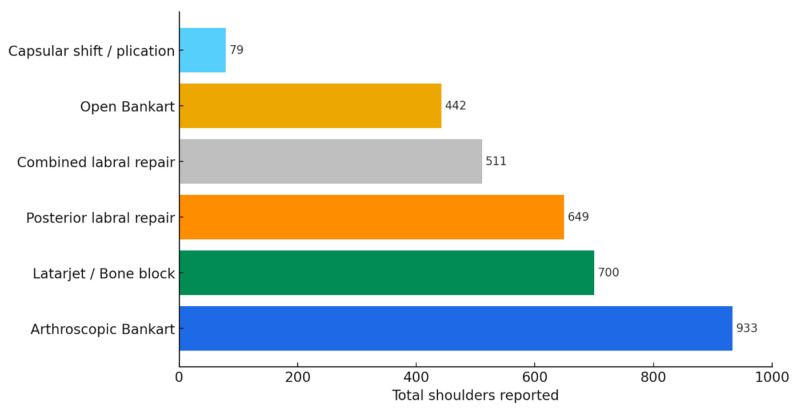
Primary stabilization procedures reported across included studies. Counts reflect shoulders and are not mutually exclusive when studies reported multiple procedure types.

Across 41 studies [7,10,15,16,17,20,23,24,25,26,27,28,29,30,31,32,33,34,35,37,40,41,42,43,44,45,46,47,49,50,51,52,53,54,55,56,58,59,60,61,62], arthroscopic Bankart repair with or without remplissage was the most frequently reported stabilization technique (18 studies; 933 shoulders) (Table 8; Figure 4) and accounted for 28.2% of shoulder stabilization procedures. Bony augmentation procedures (Latarjet, Bristow, or Eden–Hybinette) were reported in 11 studies (700 shoulders), posterior labral repair in 16 studies (649 shoulders), combined labral repair in 12 studies (511 shoulders), open Bankart repair in 4 studies (442 shoulders), and capsular shift/plication in 4 studies (79 shoulders).

Where indications were described, arthroscopic Bankart repair (with or without remplissage) was typically performed for recurrent unidirectional anterior instability in shoulders with minimal to subcritical glenoid bone loss and on-track Hill–Sachs lesions, whereas bone-augmentation procedures (e.g., Latarjet, Bristow, Eden–Hybinette) were preferentially reserved for substantial anterior glenoid bone loss, off-track bipolar lesions, or failed prior soft-tissue stabilization. Capsular shift/plication procedures were most commonly used in patients with generalized capsular laxity or multidirectional instability, and posterior capsulolabral repairs for posterior instability patterns.

#### 3.2.2. Concomitant Procedures

SLAP repair was the most frequently reported concomitant procedure (13 studies; 1689 shoulders) (Table 9). Subacromial decompression (13 studies; 1264 shoulders) and biceps tenodesis (14 studies; 1023 shoulders) were also common adjuncts. Acromioclavicular joint procedures (9 studies; 647 shoulders) and rotator cuff repair (7 studies; 309 shoulders) were less frequent, whereas rotator cuff debridement, biceps tenotomy, and other arthroscopic interventions were even less common. Of note, many shoulders underwent multiple concomitant procedures, and values are therefore not mutually exclusive.

#### 3.2.3. Failure, Recurrence, and Reoperation

Across procedure types, weighted mean failure rates (per study-reported definitions) ranged from 4.7% (posterior labral repair) to 23.6% (arthroscopic Bankart repair with or without remplissage). Complication rates were less consistently reported and ranged, on average, from 5.2% (arthroscopic Bankart repair with or without remplissage) to 10.9% (Latarjet/bone-block). Reoperation rates ranged from 5.3% (combined labral repair) to 17.7% (Latarjet/bone-block); capsular shift/plication had a 15.2% reoperation rate in a single study. Common complications included postoperative stiffness/adhesive capsulitis, infection, and transient neurologic symptoms. Outcomes are presented in Table 10.

#### 3.2.4. Return to Duty (RTD) and Return to Sport (RTS)

Reporting of functional recovery was heterogeneous. Weighted mean RTD ranged from 50.0% (open Bankart; single study) to 84.7% (posterior labral repair). Other averages included 82.0% after arthroscopic Bankart, 80.5% after combined labral repair, 78.5% after capsular shift/plication, and 63.3% after Latarjet/bone-block. RTS was infrequently reported and ranged from 4.8% (capsular shift/plication; single study) to 75.0% (combined labral repair; single study); across eight arthroscopic Bankart series, the mean RTS was 64.8%. RTD and RTS are summarized in Table 11.

## 4. Discussion

In this systematic review of U.S. active-duty servicemembers with shoulder instability, the pooled incidence of shoulder instability was 2.07 per 1000 person-years, approximately nine-fold higher than civilian estimates [8]. Across eligible cohorts, men comprised 86–96% of cases. Among studies reporting age, the mean age was 19–22 years, underscoring the predominance of young, male populations in military instability. Although anterior instability predominated, posterior and combined patterns represented a meaningful minority; across primary analyses, the pooled anterior proportion ranged from 78% to 84%. Postoperative outcomes series were dominated by arthroscopic Bankart repair, followed by bony augmentation, posterior labral repair, combined repairs, and open Bankart. Procedure-level outcomes varied: posterior labral repair demonstrated the lowest weighted failure, arthroscopic Bankart showed higher failure, and bony augmentation carried higher reoperation rates. RTD appeared highest after posterior labral repair and lower after open Bankart and bony augmentation, whereas RTS reporting was inconsistent.

Active-duty military service involves high physical demands and repetitive overhead or load-bearing activities that predispose individuals to both initial and recurrent instability, as well as to complex patterns such as posterior and combined labral tears [7,10]. Despite this burden, currently available stabilization strategies still carry a substantial risk of redislocation and reoperation in high-risk, contact populations [38,60]. Nonetheless, the operative armamentarium is evolving, with refinements in bone-augmentation techniques, remplissage, and newer dynamic stabilization concepts that aim to better address lesion-specific risk factors in these patients.

Active-duty servicemembers also present with a broad spectrum of structural pathology that directly informs operative decision-making. Anterior instability episodes may coexist with partial articular-sided rotator cuff avulsions (PASTA lesions), SLAP tears, and long-head-of-biceps pathology, particularly in overhead and throwing-specialty roles [1,6,63]. In such cases, surgeons commonly combine capsulolabral stabilization with biceps tenodesis or tenotomy and, when indicated, transtendinous or completion-and-repair treatment of PASTA lesions rather than performing isolated Bankart repair [3,59]. Conversely, collision and combat-arms servicemembers more often demonstrate recurrent bony Bankart lesions and engaging Hill–Sachs defects, for which remplissage or bone-augmentation procedures are considered [1,33,64,65,66,67,68]. Appreciating this activity- and lesion-specific distribution of pathology is essential when interpreting procedure-specific outcomes and revision risk in military cohorts.

Directionality findings warrant emphasis. Anterior instability remained more common than posterior instability, accounting for 83.9 and 16.1% respectively, yet the relative frequency of posterior and combined-type instability patterns was higher than typically emphasized among civilian populations [9,57]. Mechanistically, training modes that privilege pressing and closed-chain work, collision and combative exposures that load the shoulder in flexion/adduction/internal rotation, and fatigue from armor and ruck carriage may accentuate posterior translation and shear, predisposing to posterior labral injury and capsulolabral pathology [7,10,12,39,69]. Additionally, however, symptomatic subluxations that are disproportionately posterior as compared to frank dislocations are more likely to be evaluated and coded in military systems as posterior instability events [1,21]. The leave-one-out analysis, in which exclusion of the U.S. service academy cohort increased the anterior share to 88% with I^2^ = 0%, suggests that setting and case capture shape directionality: academy populations have narrower age ranges, high training density, mandatory sport participation, and on-site sports medicine access that may preferentially detect posterior subluxation [7,39]. Given the reduced number of eligible cohorts and heterogeneity, standardized patient-level reporting of directionality should be a priority for future surveillance.

Operative patterns reflected contemporary practice. Higher failure rates after arthroscopic Bankart repair should be interpreted cautiously, as they likely reflect differences in case mix rather than intrinsic inferiority and may be biased by the introduction of remplissage in the treatment of engaging Hill Sachs lesions. Glenoid bone loss as low as 10–16% and off-track Hill–Sachs lesions are consistently associated with increased recurrence after Bankart repair [34,48,68,70,71]. These factors are often incompletely controlled or measured in published series, confounding direct comparisons of technique effectiveness. Additional lesion- and tissue-level risk factors such as humeral avulsion of the glenohumeral ligaments (HAGL), the location and engagement pattern of Hill–Sachs defects, generalized ligamentous laxity (e.g., Beighton score), and the quality and repairability of the capsule–labrum complex are also likely to influence failure risk but were rarely reported in the included studies [72,73,74]. Consistent with these patterns, the included series generally reserved bone-augmentation and revision procedures for patients with more severe pathoanatomy, such as critical glenoid bone loss, off-track Hill–Sachs lesions, generalized capsular laxity, or prior failed stabilization, whereas arthroscopic Bankart repair with or without remplissage was typically used in shoulders with recurrent unidirectional anterior instability and limited bone loss. Where such indication gradients exist, higher revision and complication rates in bone-block or revision cohorts likely reflect underlying case severity rather than intrinsic inferiority of the procedures themselves, and lower failure rates after soft-tissue stabilization should not be overinterpreted as evidence of superiority in more complex instability patterns.

Conversely, lower failure after posterior repair may reflect clearer pathoanatomy and a lower bone-loss burden in posterior lesions, as posterior instability is less frequently associated with significant glenoid or humeral bone loss, and surgical indications are more uniform [15,36,75]. Elevated rates of reoperation after bony augmentation are consistent with indication severity and with graft- and hardware-related procedures that inherently carry revision risk; interpreting these rates as comparative effectiveness is inappropriate given that this analysis does not capture uniform bone-loss quantification, on/off-track assessment, and consistent thresholds for recurrence and revision [68,76].

Beyond the traditional dichotomy between soft-tissue Bankart repair and coracoid or free-bone augmentation, dynamic anterior stabilization (DAS) has emerged as a promising option for recurrent anterior instability with subcritical glenoid bone loss. DAS techniques transfer the long head of the biceps to the anterior glenoid to recreate a dynamic sling effect analogous to the conjoint tendon in the Latarjet procedure while preserving glenoid bone stock [77]. Early civilian series and recent systematic reviews suggest that DAS can yield favorable stability and functional outcomes in carefully selected patients with limited–to–subcritical glenoid bone loss (typically ≤20–25%), with recurrent instability rates at short- to mid-term follow-up [78,79,80]. However, none of the included U.S. military cohorts in this review reported DAS as a primary stabilization procedure, and its performance in high-risk contact and combat-arms service members remains unknown. Future military-specific studies comparing DAS with Bankart plus remplissage and bone-block procedures, stratified by glenoid bone loss and glenoid-track status, are warranted to determine whether DAS can safely bridge the gap between soft-tissue and bony reconstruction in this population.

Functional recovery requires cautious interpretation, given the infrequent and inconsistent reporting of results. RTD varied by procedure, with posterior repair generally highest and open Bankart and bony augmentation substantially lower. For counseling, clinicians should frame RTD/RTS expectations by indication severity, bone-loss pattern, occupational demands, and procedures performed. Outcomes should be documented using standardized operational definitions to enable cross-study comparability and informed disposition decisions.

Concomitant procedures were common, and SLAP repair, subacromial decompression, and biceps tenodesis were frequent adjuncts to the treatment of instability. Treating remplissage as a concomitant augmentation rather than a primary stabilization helps isolate the effect of the index stabilization but may shift observed failure and RTD if augmentation is used selectively for engaging Hill–Sachs lesions. Similar interpretive challenges are seen in civilian dynamic anterior stabilization series, where DAS is typically performed in combination with Bankart repair and, in some techniques, Hill–Sachs remplissage, yet outcomes are analyzed for the combined construct rather than the individual components [80]. Without stratified reporting, such bundled cohorts are difficult to interpret in a clinically meaningful way and would generally be judged at high risk of confounding in risk-of-bias frameworks such as ROBINS-I. Procedure-level reporting that segregates outcomes by augmentation status and quantifies glenoid bone loss and lesion tracking is needed to clarify the contribution of adjuncts in high-demand military populations.

This study is not without limitations. Incidence was summarized as a person-years–weighted fixed-effect estimate across three heterogeneous surveillance systems; heterogeneity statistics were not calculated due to inherent imprecision with very small k and differing case definitions. Directionality meta-analysis included few cohorts and substantial heterogeneity that was sensitive to cohort composition. Several outcome series lacked segregated numerators and denominators, clear primary versus revision status, or detailed reporting of concomitant procedures, limiting procedure-level pooling. Many operative series reported outcomes for which multiple procedures were performed during the same operative session without disaggregating individual components; therefore, our procedure categories often encompass bundled interventions rather than isolated techniques. This multimodal reporting further limits the strength of comparative inferences between techniques. Cadet and active-duty populations were both represented and potential overlap cannot be fully excluded. Civilian and collegiate comparators were cited only for context and were not pooled, limiting generalizability outside U.S. military settings. Some cohorts reported event-level rather than patient-level directionality, necessitating exclusion from pooling and limiting precision.

## 5. Conclusions

Shoulder instability in U.S. servicemembers occurs at rates exceeding population-based civilian estimates, with a relatively greater share of posterior and combined patterns. Operative outcomes vary across procedures and case mix.

## Figures and Tables

**Table 1:** Methodological Quality Assessment Using the MINORS Criteria.

	Balazs, 2019 [20]	Bokshan, 2021 [21]	Cameron, 2013 [22]	Casper, 2025 [23]	Chan, 2019 [24]	Chan, 2020 [25]	Christensen, 2020 [26]	Cruz, 2022 [27]	DeBerardino, 1996 [28]	Enad, 2007 [29]	Flint, 2018 [30]	Galvin, 2016 [31]	Galvin, 2017 [32]	Galvin, 2018 [33]	Green, 2022 [3]	Green, 2023 [34]	Green, 2024 [35]	Green, 2023 [36]	Hines, 2018 [37]	Kardouni, 2016 [38]	Lanzi, 2017 [39]	Lebar, 1992 [40]	McCormick, 2014 [41]	McNamara, 2025 [16]	Mescher, 2024 [42]	Min, 2023 [43]	Mologne, 1996 [44]	Mologne, 2007 [45]	Owens, 2007 [1]	Owens, 2009 [6]	Owens, 2010 [46]	Parnes, 2021 [47]	Parnes, 2024 [48]	Rodkey, 2021 [49]	Scanaliato, 2022 [17]	Scanaliato, 2025 [4]	Schroder,2006 [50]	Shaha, 2015 [51]	Slaven, 2023 [52]	Swan, 2022 [10]	Sy, 2022 [53]	Waterman, 2015 [54]	Waterman, 2016 [55]	Wolfe, 2020 [56]	Yoon, 2025 [15]	Yow, 2021 [7]
A clearly stated claim	2	2	2	2	2	2	2	2	2	2	2	2	2	2	2	2	2	2	2	2	2	2	2	2	2	2	2	2	2	2	2	2	2	2	2	2	2	2	2	2	2	2	2	2	2	2
Inclusion of consecutive patients	2	2	1	1	2	2	2	2	2	2	1	1	1	2	1	2	2	2	2	2	2	1	1	1	2	0	1	1	2	2	2	1	1	2	1	1	1	2	2	2	1	2	2	1	2	1
Prospective collection of data	2	0	2	2	0	0	0	0	0	0	0	0	0	0	0	0	0	1	0	0	1	1	2	0	1	0	0	0	2	2	2	2	1	1	0	0	0	1	1	1	0	0	0	0	0	0
Endpoints appropriate to the aim of the study	2	2	2	2	2	2	2	2	2	2	2	2	2	2	2	2	2	2	2	2	2	2	2	2	2	2	2	2	2	2	2	2	2	2	2	2	2	2	2	2	2	2	2	2	2	2
Unbiased assessment of the study endpoints	1	1	2	1	1	1	1	1	1	1	1	2	1	1	1	1	1	1	1	2	1	1	2	1	1	1	1	1	1	2	2	1	1	1	1	1	1	1	1	2	1	2	2	1	1	1
Follow-up period appropriate to the aim of the study	2	0	2	1	2	2	2	2	2	2	2	2	2	2	2	2	2	2	2	2	2	2	2	2	2	2	2	2	2	2	2	2	2	2	2	2	2	2	2	2	2	1	2	2	2	2
Loss to follow-up less than 5%	1	0	1	0	1	1	2	1	2	0	2	2	0	2	2	2	1	1	0	2	1	1	1	1	1	0	1	1	1	1	1	1	1	1	2	2	1	1	2	2	1	1	1	1	1	1
Prospective calculation of the study size	0	0	0	2	0	0	0	0	0	0	0	0	0	0	0	0	0	0	1	0	0	0	2	0	0	0	0	0	0	0	0	0	0	0	0	0	0	0	0	0	0	0	0	0	0	0
Additional criteria in the case of comparative study																																														
An adequate control group	0		2	2	2	0	0					2	2			2	2		1							2												2							2	
Contemporary groups	0		2	2	2	0	0					2	2			2	2		2							2												2							2	
Baseline equivalence of groups	0		1	2	1	0	0					1	2			1	1		1							2												1							1	
Adequate statistical analyses	1		2	2	2	1	1					2	2			2	2		2							2												2							2	
Maximum possible score	24	16	24	24	24	24	24	16	16	16	16	24	24	16	16	24	24	16	24	12	16	16	16	16	16	24	16	16	16	16	16	16	16	16	16	16	16	24	16	16	16	16	16	16	24	16
Total MINORS score	13	7	19	19	17	11	12	10	11	9	10	18	16	11	10	18	17	11	16	16	11	10	14	9	11	17	9	9	12	13	13	11	10	11	10	10	9	18	12	13	9	10	11	9	17	9

**Table 2:** Baseline Characteristics of Eligible Epidemiologic Studies.

Study (Year)	Level of Evidence	Study Design	Military Population Represented	Years of Data Collection	Setting/Data Source
Owens et al. (2009) [6]	III	Retrospective cohort	U.S. military (all branches)	1998–2006	Defense Medical Epidemiology Database (DMED)
Owens et al. (2007) [1]	II	Prospective cohort	U.S. Military Academy (cadets)	2004–2005	US Military Academy (USMA)
Cameron et al. (2013) [22]	II	Prospective cohort	U.S. Military Academy (cadets)	2006–2010	US Military Academy (USMA)
Lanzi et al. (2017) [39]	III	Retrospective cohort	U.S. Military Academy (cadets)	2006–2012	US Military Academy (USMA)
Kardouni et al. (2016) [38]	III	Retrospective cohort	U.S. Army (active-duty)	2002–2011	US Army medical encounter data
Yow et al. (2021) [7]	III	Retrospective cohort	U.S. Naval Academy (clinical records)	2009–2020	US Naval Academy (USNA)
Bokshan et al. (2021) [21]	III	Retrospective cohort	U.S. military (all branches)	2016–2018	Defense Medical Epidemiology Database (DMED)
Swan et al. (2022) [10]	III	Retrospective cross-sectional	U.S. military (all branches, operative cases)	2016–2019	Wounded, Ill, and Injured Registry (DoD)

**Table 3:** Epidemiologic Findings from Studies of Shoulder Instability among U.S. Military Cohorts.

Study (Year)	Military Population	Years of Data Collection	Events	Person-Years	Incidence (/1000 PY)	Instability Type(s)
Military population						
Owens et al. (2009) [6]	U.S. military (all branches)	1998–2006	19,730	11,680,893	1.69	Dislocation (overall)
Owens et al. (2007) [1]	U.S. Military Academy (cadets)	2004–2005	-	-	2.8% ^†^	Traumatic instability
Kardouni et al. (2016) [38]	U.S. Army (active-duty)	2002–2011	15,426	4,923,463	3.13	Dislocation (overall)
Bokshan et al. (2021) [21]	U.S. military (all branches)	2016–2018	7154	3,868,007	1.84	All instability
Pooled Military	-	1998–2019	42,310	20,472,363	2.07 (95% CI, 2.05–2.09)	
Civilian and collegiate reference						
Zacchilli et al. (2010) [8]	National Electronic Injury Surveillance System (NEISS)	2002–2006	8940	-	0.24	Dislocation (overall)
Owens et al. (2009) [5]	NEISS (athletes)	1989–2004	4080	32,843,226 *	0.12 *	All instability

^†^ Value reported as 1-year incidence proportion, not per 1000 person-years. * Athlete denominators are per 1000 athlete exposures and are not directly comparable to person-years (PY). PY = person-years; - = not reported.

**Table 4:** Demographic characteristics and reported risk factors for shoulder instability among U.S. military cohorts.

Study (Year)	Military Population	Years of Data Collection	Mean Age (Years)	Male (%)	Reported Risk Factors
Owens et al. (2009) [6]	U.S. military (all branches)	1998–2006	-	92	Male sex, White race, Army service, junior enlisted rank, age < 30
Kardouni et al. (2016) [38]	U.S. Army (active-duty)	2002–2011	-	86	Male sex, age ≤ 35, White race, education level, concurrent axillary nerve injury
Cameron et al. (2013) [22]	U.S. Military Academy (cadets)	2006–2010	18.8 ± 1.0	88	Prior instability history
Yow et al. (2021) [7]	U.S. Naval Academy (clinical records)	2009–2020	22 ± 4	88	-
Bokshan et al. (2021) [21]	U.S. military (all branches)	2016–2018	-	96	Male sex, age 30–34, White race, enlisted rank, service branch

- = not reported. Predictors listed only when identified in adjusted models or when reported with an incidence rate ratio or odds ratio and a 95% confidence interval excluding 1.0 (per prespecified rule).

**Table 5:** Distribution of Instability Direction Categories by Cohort in U.S. Military Populations.

Study (Year)	Military Population	Years of Data Collection	Total Cases of Instability (*n*)	Anterior *n* (%)	Posterior *n* (%)	Combined *n* (%)	Multidirectional *n* (%)
**Military population**							
Owens et al. (2007) [1]	U.S. Military Academy (cadets)	2004–2005	117	94 (80.3)	12 (10.3)	—	11 (9.4)
Cameron et al. (2013) [22] ^†^	U.S. Military Academy (cadets)	2006–2010	46	39 (84.8)	7 (15.2)	—	—
Lanzi et al. (2017) [39]	U.S. Military Academy (cadets)	2006–2012	633	457 (72.1)	63 (17.9)	—	—
Yow et al. (2021) [7]	U.S. Naval Academy (clinical records)	2009–2020	443	210 (47.4)	80 (18.1)	153 (34.5)	—
Bokshan et al. (2021) [21]	U.S. military (all branches, posterior only)	2016–2018	—	—	372 (5.2)	—	—
Swan et al. (2022) [10]	U.S. military (all branches, operative cases)	2016–2019	311	94 (30.2)	76 (24.4)	136 (43.7)	—
**Civilian and Sport-related population reference**							
Shields et al. (2017) [9]	General Population, U.K. Trauma Database	2002–2006	329	—	2.7%	—	—
Javed et al. (2019) [57]	Consecutive Surgical Records (sporting vs nonsporting)	2012–2014	Sporting: 331 Nonsporting: 111	Sporting: 158 (47.7) Nonsporting: 76 (68.5)	Sporting: 58 (17.5) Nonsporting: 14 (12.6)	Sporting: 115 (34.7) Nonsporting: 21 (18.9)	—

Proportions reflect study-specific reporting. Where studies listed additional categories (e.g., isolated superior labral in [7]), percentages may not sum to 100%. — = not reported. ^†^ Event-level dataset, not directly comparable.

**Table 6:** Distribution of Unidirectional Shoulder Instability among U.S. Military Cohorts.

Instability Direction	Pooled % (95% CI)	Range Across Studies (%)	Unidirectional *n*	Cohorts Included
**Epidemiology cohorts only**				
Anterior	83.9 (70.5–91.9)	72.4–88.7	916	[6,7,8]
Posterior	16.1 (8.1–29.5)	11.3–27.6	916	[6,7,8]
**Sensitivity: operative cohort included**				
Anterior	78.4 (60.8–89.5)	55.3–88.7	1086	[5,6,7,8]
Posterior	21.6 (10.5–39.2)	11.3–44.7	1086	[5,6,7,8]

Random-effects model (logit-transformed proportions; inverse-variance weighting). Unit of analysis is patient-level, unidirectional cases; event-level datasets and one-sided reports were excluded. Within-study proportions were recalculated so anterior + posterior = 100% of unidirectional cases. Study counts: epidemiology-only meta-analysis k = 3 (*n* = 916); sensitivity including the operative-only cohort k = 4 (*n* = 1086). Heterogeneity: I^2^ = 93.9% (epidemiology only) and I^2^ = 96.5% (sensitivity including operative cohort). CI = Confidence Interval.

**Table 7:** Primary vs. Recurrent Presentation and Recurrence During Follow-up among U.S. military cohorts.

Study (Year)	Military Population	Years of Data Collection	Population at Risk (*n*); Instability Events (*n*)	Follow-Up Window	Index Presentation: Primary	Index Presentation: Recurrent	Recurrence During Follow-Up (%)	Definition of Recurrence
Owens et al. (2007) [1]	U.S. Military Academy (cadets)	2004–2005	4141 cadets; 117 incident instability events	Cross-sectional (one academic year); no longitudinal follow-up	Subluxation: 45.5% Dislocation: 66.7	Subluxation: 54.5%; Dislocation: 33.3%	-	Not assessed longitudinally; study captured incident events within a single academic year
Kardouni et al. (2016) [38]	U.S. Army (active-duty)	2002–2011	1,261,297 soldiers; 15,426 incident dislocations	2 years after index	-	-	28.7	Repeat dislocation or instability episode coded ≥3 months and ≤2 years after the index event

Percentages for index presentation in Owens et al. (2007) [1] are within event type and therefore do not sum to 100% across all instability events. - = not reported.

**Table 8:** Primary Stabilization Procedures Reported among U.S. Military Cohorts.

Procedure Type	No. of Studies Reporting	Total Shoulders Performed
Arthroscopic Bankart	18	933
Latarjet/Bone block	11	700
Posterior labral repair	16	649
Combined labral repair	12	511
Open Bankart	4	442
Capsular shift/plication	4	79

Counts reflect shoulders reported for each procedure type; studies reporting multiple procedures contribute to more than one category.

**Table 9:** Concomitant Procedures Performed at the Time of Index Shoulder Stabilization among U.S. Military Cohorts.

Procedure Type	No. of Studies Reporting	Shoulders Receiving Procedure (*n*)
SLAP repair	13	1689
Subacromial decompression	13	1264
Biceps tenodesis	14	1023
AC (acromioclavicular) joint procedures	9	647
Rotator cuff repair	7	309
Remplissage	5	41
Rotator cuff debridement	7	32
Biceps tenotomy	1	3

Counts reflect shoulders receiving each concomitant procedure during the same operative episode as the primary stabilization; studies could contribute to multiple categories, so totals are not mutually exclusive and may exceed the number of stabilized shoulders.

**Table 10:** Reported Outcomes Following Shoulder Stabilization among U.S. Military Cohorts.

Procedure	No. of Studies	Follow-Up (Months), Mean ± SD (Range)	Failure %, Mean ± SD (Range)	Complications %, Mean ± SD (Range)	Reoperation %, Mean ± SD (Range)	Common Complications
Arthroscopic Bankart	18	50.5 ± 28.5 (24.0–106.9)	23.6 ± 13.9 (9.8–45.5)	5.2 ± 6.8 (0.0–14.3)	10.4 ± 7.7 (0.0–18.4)	Infection, postoperative stiffness, transient nerve injury
Open Bankart	4	44.6 ± 167.3 (26.0–316.8)	13.3 ± 17.9 (8.0–33.3)	-	-	-
Latarjet/Bone block	11	82.6 ± 96.3 (2.4–316.8)	13.4 ± 5.4 (8.7–20.0)	10.9 ± 11.1 (8.7–32.2)	17.7 ± 9.2 (14.0–30.5)	Neurologic injury, graft fracture, infection
Capsular shift/plication	4	34.0 *	13.5 ± 2.6 (10.6–14.3)	-	15.2 *	-
Posterior labral repair	16	55.8 ± 26.5 (35.0–106.9)	4.7 ± 2.7 (1.5–8.1)	7.1 ± 8.5 (0.0–20.0)	6.2 ± 3.8 (0.0–8.1)	Postoperative stiffness/adhesive capsulitis
Combined labral repair	12	73.2 ± 32.7 (35.0–140.4)	10.0 ± 22.4 (5.8–37.5)	-	5.3 ± 2.7 (0.0–5.8)	Global stiffness

- = not reported. SD = standard deviation. Failure includes recurrent instability (dislocation or subluxation) or reoperation for instability. Reoperation includes any unplanned return to the operating room after index stabilization. Means are weighted by study sample size where available; SD reflects between-study variability. * Reported by only one study.

**Table 11:** Return-to-Duty (RTD) and Return-to-Sport (RTS) after Primary Shoulder Stabilization among U.S. military Cohorts.

Procedure	No. of Studies	RTD %, Mean ± SD (Range)	RTS %, Mean ± SD (Range)
Arthroscopic Bankart	8	82.0 ± 15.7 (56.0–93.9)	64.8 ± 41.2 (4.8–95.1)
Open Bankart	1	50.0 *	-
Latarjet/Bone block	4	63.3 ± 34.0 (14.3–89.1)	10.3 *
Capsular shift/plication	2	78.5 ± 10.3 (71.2–85.7)	4.8 *
Posterior labral repair	9	84.7 ± 11.9 (58.1–95.9)	-
Combined labral repair	8	80.5 ± 16.4 (56.0–93.3)	75.0 *

- = not reported. SD = standard deviation. Means are weighted by study sample size where available; SD reflects between-study variability. RTD was defined as return to unrestricted full duty at the same or higher level of responsibility; RTS as return to the preinjury (or higher) sport level. * Reported by only one study.

## Data Availability

The raw data supporting the conclusions of this article will be made available by the authors on request.

## Supplementary Materials

The following supporting information can be downloaded at: https://www.mdpi.com/article/10.3390/jcm15010110/s1, PRISMA Checklist.
